# Engineering Human 3D Cardiac Tissues for Predictive Functional Drug Screening

**DOI:** 10.3390/pharmaceutics18010018

**Published:** 2025-12-22

**Authors:** Ester Sapir Baruch, Daniel Rosner, Elisabeth Riska, Moran Yadid, Assaf Shapira, Tal Dvir

**Affiliations:** 1 The Shmunis School of Biomedicine and Cancer Research, Faculty of Life Sciences, Tel Aviv University, Tel Aviv 6997801, Israel; estersapirbaruch@gmail.com (E.S.B.);; 2 Department of Materials Science and Engineering, Faculty of Engineering, Tel Aviv University, Tel Aviv 6997801, Israel; 3 The Center for Nanoscience and Nanotechnology, Tel Aviv University, Tel Aviv 6997801, Israel; 4 Sagol Center for Regenerative Biotechnology, Tel Aviv University, Tel Aviv 6997801, Israel; 5 Sagol School for Neuroscience, Tel Aviv University, Tel Aviv 6997801, Israel; 6 School of Biomedical Engineering, Faculty of Engineering, Tel Aviv University, Tel Aviv 6997801, Israel

**Keywords:** cardiotoxicity, hiPSC-derived cardiac tissue, 3D in vitro model, drug screening

## Abstract

**Background/Objectives**: Cardiotoxicity remains a leading cause of drug withdrawal. Conventional preclinical models, such as two-dimensional (2D) cell cultures and animal studies, often fail to accurately predict human cardiac responses. While 2D cultures lack the complex architecture and dynamic functionality of native myocardium, interspecies differences limit the translational relevance of animal models. The objective of this study was to develop a human-relevant, in vitro platform that enables predictive and functional assessment of drug-induced cardiotoxicity. **Methods**: Here, we present a high-throughput in vitro platform for cardiotoxicity screening using three-dimensional (3D) cardiac tissues derived from human induced pluripotent stem cells (hiPSCs) within a thermoresponsive extracellular matrix-derived hydrogel. The hydrogel enables homogeneous encapsulation, differentiation in 3D, and long-term assembly into a functional cardiac tissue. Maturation was validated by immunostaining for cardiac-specific markers, and calcium imaging was employed to monitor electrical signal propagation. Contractile performance, defined by beat rate and contraction amplitude, was quantified using video-based motion analysis. The platform was applied to evaluate the dose-dependent effects of various cardioactive compounds, including β-adrenergic agonists ((-) epinephrine and dopamine), a cardiotoxic chemotherapeutic (doxorubicin), a sinus node inhibitor (ivabradine), a calcium channel blocker (verapamil), and a β-adrenergic antagonist (metoprolol). **Results**: The engineered cardiac tissues exhibited functional maturation and stable contractile behavior. Drug testing demonstrated compound-specific, dose-dependent functional responses. For each compound, the system faithfully reproduced the expected physiological responses. **Conclusions**: This human-relevant, scalable platform enables sensitive, multiparametric functional assessment of cardiac tissues, offering a cost-effective and predictive tool for preclinical drug safety testing. By bridging the gap between in vitro assays and human physiology, it holds promise to enhance translational accuracy while reducing reliance on animal models.

## 1. Introduction

Cardiotoxicity is the deleterious effect of pharmaceutical compounds on cardiac structure and function and is a leading cause of drug attrition in late-stage clinical trials and post-market withdrawal. Preclinical approaches largely rely on two-dimensional (2D) cell cultures and animal models, both of which suffer from translational limitations and ultimately pose a significant hurdle to the development of new therapeutics, contributing to increased costs, delayed approval timelines, and risks to patient safety [1]. Consequently, effective preclinical models are crucial for identifying cardiotoxic liabilities early in the drug development pipeline.

Two-dimensional (2D) cultures of cardiomyocytes, while widely used due to their availability and cost-effectiveness, lack the three-dimensional (3D) architecture, cellular heterogeneity, and extracellular matrix (ECM) interactions of native cardiac tissue [2]. This simplification results in altered cellular behaviors, including gene expression, electrophysiology, and mechanical function, limiting their ability to model complex tissue responses and chronic drug effects [3,4]. Animal models offer a higher degree of physiological complexity but exhibit interspecies variations in cardiac electrophysiology, receptor distribution, metabolism, and immune responses that frequently impair their predictive value for human outcomes [5,6,7]. For example, differences in ion channel expression and β-adrenergic receptor subtypes between rodents and humans can lead to divergent drug responses, complicating extrapolation of preclinical findings [6,7]. These challenges are reflected in the high attrition rates of drug candidates due to unforeseen cardiotoxicity and emphasize the need for improved, human-relevant in vitro platforms [8,9]. In line with this shift, the Food and Drug Administration (FDA) has recently published a formal “Roadmap to Reducing Animal Testing in Preclinical Safety Studies”, signaling a strategic shift toward replacing reliance on animal studies with human-based in vitro, organ-on-a-chip, and computational models (so-called New Approach Methodologies, NAMs) [10]. By endorsing the regulatory use of NAM-based evidence, the FDA is redefining the role of animal testing in preclinical safety studies, positioning it as an auxiliary measure rather than the default standard.

3D cardiac tissues derived from human induced pluripotent stem cells (hiPSCs) have emerged as promising models to bridge this translational gap. These multicellular constructs self-organize to recapitulate aspects of myocardial tissue architecture, including cell–cell and cell–ECM interactions, electrophysiological coupling, and synchronized contractility [11,12,13]. Numerous engineered heart tissue (EHT) and organoid approaches, including dynamic-loading maturation systems, scaffold-based assemblies, perfusable microfluidic tissues, and bio-fabrication strategies, have demonstrated that 3D culture enhances structural and functional maturation compared to traditional 2D monolayers [14,15,16,17,18,19,20]. However, most existing platforms rely on pre-differentiated hiPSC-cardiomyocytes that are subsequently incorporated into synthetic or reconstituted matrices, requiring multistep workflows and often resulting in heterogeneous cellular integration.

To address these limitations, our group has developed a thermoresponsive, ECM-derived hydrogel that enables direct 3D differentiation of hiPSCs into cardiomyocytes within a fully biological, native-like matrix, allowing for maturation periods exceeding 30 days [11,12,13]. This hydrogel remains liquid at room temperature, facilitating uniform cell encapsulation, and solidifies at physiological temperature to support 3D tissue formation. The resulting cardiac tissues develop organized sarcomeric structures and exhibit spontaneous beating activity. These engineered constructs display robust electrophysiological properties, making them well suited for functional characterization and drug screening applications.

Previous studies employing 3D microtissues for cardiotoxicity often include validation solely using doxorubicin as a benchmark for model accuracy [15,21,22,23,24,25,26]. However, the mechanism of action for the cardiotoxicity of doxorubicin is not well understood and requires chronic exposure or unrealistic dosage. Therefore, the use of doxorubicin as an exclusive predictor of a model’s physiological relevance is not sufficient [24]. Some studies that test a broader variety of compounds fail to capture the developmental and microenvironmental context of native cardiac tissue. In these models, cardiomyocytes are typically generated in 2D and combined with non-personalized scaffold material immediately prior to compound exposure [27,28].

**Figure 1:**
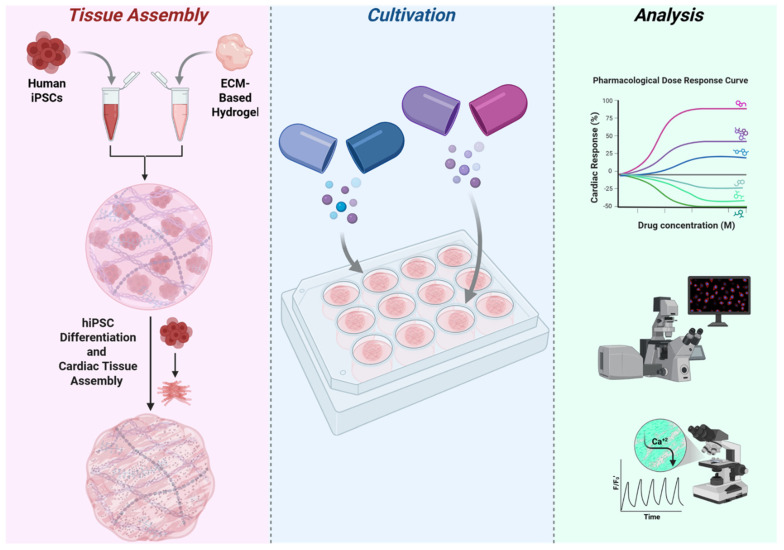
Overview of the 3D hiPSC-derived cardiac tissue platform for cardiotoxicity screening. Human induced pluripotent stem cells (hiPSCs) are encapsulated within a thermoresponsive ECM-based hydrogel and undergo differentiation into cardiomyocytes in a 3D microenvironment. The tissues are cultivated for 30 days and subsequently exposed to varying drug concentrations to generate dose–response curves based on changes in heart rate. At concentrations causing a 50% increase or decrease in beat rate (Heart Beat, 50), further functional analyses are performed, including video-based motion analysis of contraction amplitude, calcium imaging of calcium transient propagation, and immunofluorescence staining for cardiac-specific markers to assess tissue maturation and structure. This figure was created in BioRender. Baruch, S. (2026) https://BioRender.com/gy0n8im.

In the present study, we aim to develop and validate a scalable, high-throughput platform for cardiotoxicity screening using hiPSC-derived cardiac tissue within this hydrogel system. To comprehensively assess cardiotoxic effects, we employed video-based motion analysis and calcium imaging to measure beat rate, contraction amplitude, and calcium transient propagation, key functional parameters reflecting myocardial contractility and electrical activity. A graphical summary illustrating the development and application of this platform is provided in Figure 1. We evaluated six pharmacologically diverse compounds targeting key cardiac mechanisms. Each compound was predicted to affect beat rate, contraction amplitude, and calcium transient propagation in distinct ways. For example, β-adrenergic agonists ((-) epinephrine and dopamine) stimulate β1-adrenergic receptors, which increase intracellular cAMP and calcium influx, resulting in increased beat rate (positive chronotropy), enhanced contraction amplitude (positive inotropy), and accelerated calcium transient propagation [29]. In contrast, doxorubicin, a chemotherapeutic agent with known cardiotoxicity, induces oxidative stress and mitochondrial dysfunction that impair calcium handling, leading to decreased contraction amplitude, slowed and irregular calcium transients, and potentially arrhythmic beat patterns [30]. Ivabradine selectively inhibits the “funny” current (If) in sinoatrial node cells, resulting in a reduced beat rate (negative chronotropy) without significantly affecting contraction amplitude or calcium transient characteristics [31]. Verapamil, a calcium channel blocker, reduces L-type calcium current, causing decreased calcium influx during excitation–contraction coupling, which manifests as reduced contraction amplitude (negative inotropy) and slower calcium transient propagation with a moderate decrease or no change in beat rate [29]. Metoprolol, a β-adrenergic antagonist, blocks sympathetic β1-receptors, reducing heart rate (negative chronotropy), contraction amplitude (negative inotropy), and dampening calcium transient amplitude and speed by inhibiting β-adrenergic signaling pathways [32]. A summary of the compounds and their effect on heart tissues is shown in Table 1.

Our findings demonstrate that this 3D tissue-based platform can detect drug-specific alterations in cardiac function, highlighting its potential as a physiologically relevant, multiparametric, and human-specific tool for evaluating preclinical cardiotoxicity. By improving predictive accuracy and scalability, this system could reduce dependence on animal models and accelerate the development of safer cardiovascular and systemic therapeutics.

## 2. Materials and Methods

**Preparation of ECM-Based Hydrogel.** Porcine omental tissue was sourced from Kibbutz Lahav, Israel, and processed according to established decellularization protocols [11,12,13]. Briefly, tissue was washed with phosphate-buffered saline (PBS), large vessels were excised, and samples were immersed in hypotonic buffer (10^−3^ M Tris, 5^−3^ M EDTA, 1^−6^ M PMSF, pH 8) for 1 h at room temperature, followed by three freeze–thaw cycles (−80 °C). Samples were sequentially washed for 30 min in 70% ethanol, 96% ethanol, and acetone (three times), and then soaked for 24 h in 40% acetone in n-hexane with three solution changes. After an additional 30 min wash in 96% ethanol and overnight incubation at 4 °C in 70% ethanol, tissue underwent four PBS washes and was incubated overnight in 0.25% trypsin-EDTA. This was followed by four PBS washes and 24 h in 1.5 M NaCl with three solution changes. Tissue was treated for 1 h in 50^−3^ M Tris (pH 8) containing 1% Triton X-100, then washed thoroughly with PBS and double-distilled water (DDW), frozen (−20 °C), lyophilized, and milled into flakes. The dry extracellular matrix (dECM) was dissolved in 0.1 M HCl (1.67% *w*/*v*) and enzymatically digested with porcine pepsin (1 mg per 10 mg dECM) for 3–4 days at room temperature. Following digestion, pH was adjusted to 7.4 using 5 M NaOH, and 10× PBS was added to reach a 1× final concentration (final dECM concentration 1.5% *w*/*v*). The solution was filtered through a sterile 70 μm nylon mesh and supplemented with 0.1% penicillin–streptomycin.

**High-Resolution Scanning Electron Microscopy (hrSEM).** Samples were fixed in 2.5% glutaraldehyde for 2 h at RT, dehydrated through graded ethanol (50–100% *v*/*v*), and dried using critical point drying (Balzers, Balzers, Liechtenstein). Dried samples were gold sputter-coated (Polaron E 5100, Quorum Technologies, Lewes, UK) and imaged with a Gemini 300 hrSEM (Zeiss, Oberkochen, Germany).

**Rheological Properties.** Rheological measurements (n = 3) were conducted using a Discovery HR-3 Hybrid Rheometer (TA Instruments, New Castle, DE, USA) equipped with parallel plate geometries (20 mm diameter) and a Peltier plate for precise temperature control. To investigate the gelation kinetics of ECM-based hydrogel, time sweep measurements were performed at 37 °C for 2000 s using a constant frequency of 1 Hz and 1% strain, with initial loading at 20 °C.

**Human iPSC Culture.** Human induced pluripotent stem cells (iPSCs) were generated from omental stromal cells and kindly provided by Dr. Rivka Ofir (Ben Gurion University, Israel; cell lines BGUi013-A and BGUi014-A, registered at https://www.hpscreg.eu/cell-line/BGUi013-A, accessed on 27 April 2022 and https://www.hpscreg.eu/cell-line/BGUi014-A, accessed on 4 February 2021). Undifferentiated iPSCs were cultured on 10 cm tissue culture plates (Corning, Corning, NY, USA) pre-coated with Matrigel^®^ (Corning, Bedford, MA, USA) diluted to 250 µg mL^−1^ in DMEM/F12 medium (Sartorius Israel Ltd., Beit HaEmek, Israel). Cells were maintained in NutriStem^®^ hPSC XF medium (Sartorius Israel Ltd., Beit HaEmek, Israel) supplemented with 0.1% penicillin–streptomycin (Sigma-Aldrich, Rehovot, Israel) in a humidified incubator at 37 °C with 5% CO_2_. Medium was refreshed daily, and cells were passaged at 80% confluence using ReLeSR™ (StemCell Technologies, Vancouver, BC, Canada) according to the manufacturer’s instructions.

**Cardiomyocyte Differentiation in 3D.** iPSCs were encapsulated directly into the ECM hydrogel and differentiated in 3D following our previously established ECM-based encapsulation frameworks [13,38,39,40,41]. This method supports spontaneous emergence of heterogeneous cardiac populations, including pacemaker-like, atrial-like, and ventricular-like phenotypes, as commonly reported for 3D iPSC differentiation systems. Briefly, Growth medium (NutriStem™, Biological Industries, Beit HaEmek, Israel) was refreshed daily until induced pluripotent stem cells (iPSCs) reached 80% confluence in 10 cm culture plates. Undifferentiated cells were dissociated using 1 U·mL^−1^ dispase (StemCell Technologies, Vancouver, BC, Canada), followed by gentle mechanical trituration. The resulting cell suspension was homogenously mixed with 1.5% (*w*/*v*) omentum-derived hydrogel. Three-microliter droplets containing small iPSC colonies embedded within extracellular matrix (ECM)-based hydrogel were dispensed onto untreated 10 cm Petri dishes. The droplets were incubated at 37 °C for 45 min to induce physical crosslinking, forming stable 3D implants. Culture medium (NutriStem™) was refreshed daily until the embedded iPSCs reached full confluence within 1–3 days. On day 0, the medium was replaced with RPMI 1640 (Biological Industries) supplemented with 0.5% L-glutamine (Biological Industries), B27 minus insulin (50×, Invitrogen, Carlsbad, CA, USA), and 10 µM CHIR-99021 (Tocris, Bristol, UK). Medium was refreshed every other day. After 36 h, CHIR-99021 was withdrawn, and on day 3, 5 µM IWP (Wnt processing inhibitor, Tocris) was added for 48 h, followed by removal on day 5. By day 7, spontaneous contractile activity was observed. At this point, medium was replaced with RPMI 1640 supplemented with 0.5% L-glutamine, B27 minus retinoic acid (50×, Invitrogen), and 1 µM retinoic acid (Sigma-Aldrich, Darmstadt, Germany). From day 11 onwards, cultures were maintained in M-199 medium (Biological Industries) containing 500 U·mL^−1^ penicillin, 100 µg·mL^−1^ streptomycin, 5% fetal bovine serum (Biological Industries), 0.6^−3^ M CuSO_4_·5H_2_O, 0.5^−3^ M ZnSO_4_·7H_2_O, and 1.5^−3^ M vitamin B12 (Sigma-Aldrich). This medium was refreshed every other day.

**Immunofluorescence Staining.** Samples were fixed with 3.5% formaldehyde (Bio-Lab, Jerusalem, Israel) for 30 min at room temperature (RT), then washed three times with phosphate-buffered saline (PBS). Permeabilization was performed using 0.1% (*v*/*v*) Triton X-100 (Sigma-Aldrich, Darmstadt, Germany) for 10 min, followed by blocking in 2% bovine serum albumin (BSA; MP Biomedicals, Santa Ana, CA, USA) in PBS for 1 h at RT. Samples were incubated overnight at 4 °C with primary antibodies diluted in 2% BSA blocking solution (see Antibodies list). After three PBS washes, samples were incubated for 1.5 h at RT with fluorophore-conjugated secondary antibodies diluted in 2% BSA. Nuclear staining was performed simultaneously using DAPI (ready-made solution, 1:50; Sigma-Aldrich). Imaging was conducted on a confocal microscope (Nikon Eclipse NI-E, Nikon Instruments, Melville, NY, USA), and images were processed using NIS-Elements BR 3.2 software (Nikon).

**Antibodies List.** Antibodies for stem cells: Mouse αOct3/4 (IgG2b) (SC 5279) 1:250 (Santa-Cruz, Santa Cruz, CA, USA). Rabbit to Ki67 (ab16667), 1:250 (Abcam, Boston, MA, USA). Antibodies for cardiac cells: Rabbit to Sarcomeric Alpha Sarcomeric Actinin (ab68167), 1:200 (Abcam). Rabbit to Connexin 43 Intercellular Junction (ab11370), 1:400 (Abcam). Secondary antibodies: Goat Anti-Rabbit (Alexa Fluor 488) (ab2338046), 1:250 (Jackson ImmunoResearch, PA, USA). Goat Anti-Mouse (Alexa Fluor 555) (ab150118), 1:500 (Abcam). Goat Anti-Mouse (Alexa Fluor 647) (ab2338902), 1:250 (Jackson ImmunoResearch). For detection of nuclei, cells were incubated with DAPI ready-made solution, 1:50 (Sigma-Aldrich).

**Functional Drug Screening in hiPSC-Derived Cardiac Tissues via Calcium Imaging and Motion Analysis.** Cardiac iPSC implants were imaged using an inverted fluorescence microscope (Nikon Eclipse Ti-E, Nikon Instruments, Melville, NY, USA). Image processing and analysis were performed with NIS-Elements software BR 3.2 (Nikon Instruments) and ImageJ 2.17.0 (FIJI). Representative data were collected from three different implants per experiment (n = 3). For calcium imaging, implants were incubated with 860 µM Fluo-4 AM (Invitrogen, Carlsbad, CA, USA) and 3.6% Pluronic F-127 (Sigma-Aldrich, Darmstadt, Germany), diluted 1:25 (*v*/*v*) in Hanks’ Balanced Salt Solution (HBSS; Biological Industries, Beit HaEmek, Israel) for 45 min at 37 °C. Following incubation, implants were exposed to six pharmacological agents (all purchased from Sigma-Aldrich): (-)-epinephrine (10^−9^ M to 10^−5^ M), verapamil (10^−8^ M to 10^−4^ M), metoprolol (10^−6^ M to 10^−2^ M), doxorubicin (10^−7^ M to 10^−3^ M), dopamine (10^−8^ M to 10^−4^ M), and ivabradine (10^−8^ M to 10^−4^ M)—with 10 min incubation at 37 °C for each concentration prior to imaging. Calcium transients were recorded at 100 frames per second using an ORCA-Flash 4.0 digital CMOS camera (Hamamatsu Photonics, Hamamatsu, Japan) attached to the microscope. Image sequences were processed in ImageJ (FIJI) to filter calcium signal data, and spatiotemporal heat maps were generated in MATLAB R2024b (MathWorks, Natick, MA, USA) (MathWorks, Natick, MA, USA) using a custom script to identify the time point of maximum fluorescence intensity change for each pixel. Activation times were extracted from the temporal derivative of fluorescence intensity, and isochronal activation maps were constructed based on the spatial distribution of activation delays. Although calcium imaging provides an indirect approximation of conduction velocity, it is widely used to evaluate electrical propagation in engineered heart tissues and iPSC-derived 3D constructs, particularly when voltage-sensitive dyes or MEA-based measurements are impractical due to tissue thickness or optical constraints [28,42,43]. Accordingly, conduction patterns in this study were interpreted qualitatively and semi-quantitatively based on activation gradients rather than absolute CV values. Implants were similarly imaged under brightfield conditions using the Nikon Eclipse Ti microscope. Videos were acquired at 100 frames per second with the same ORCA-Flash 4.0 camera (Hamamatsu Photonics, Hamamatsu, Japan) setup. Contractile amplitude was quantified through video-based motion analysis performed on implants exposed to drug concentrations that altered the beat rate by ±50%.

**Statistical Analysis.** All statistical analyses were performed on biological replicates (n = 3 per group unless otherwise indicated). Statistical analyses were presented as mean ± standard error of mean on the basis of at least three replicates. Differences between samples were assessed by the relevant tests (details for each experiment were provided alongside the data), and *p* < 0.05 was considered significant. Normalization procedures are described within each figure legend. Analyses were performed using GraphPad Prism 8 (Version 8.4.2) for Windows (GraphPad Software).

## 3. Results

### 3.1. Preparation and Characterization of ECM-Based Hydrogel

**Figure 2:**
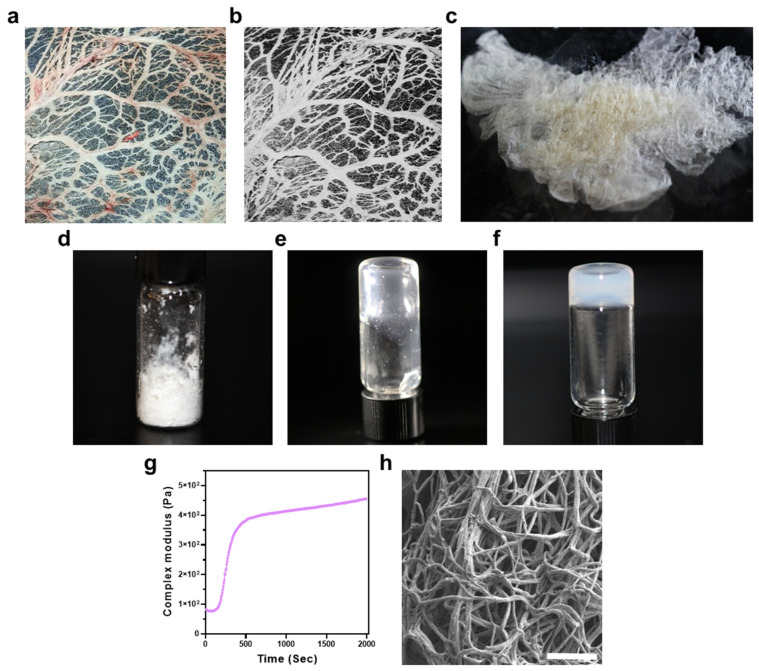
Characterization of the ECM-based hydrogel. (**a**) Native omental tissue with intact vascularized structure. (**b**) Translucent omental tissue post-decellularization. (**c**) Dried, decellularized ECM. (**d**) ECM milled into fine powder. (**e**) Enzymatically digested ECM pre-gel solution at room temperature. (**f**) Thermally induced gelation at 37 °C. (**g**) Rheological analysis of gelation kinetics, indicating rapid modulus increase upon warming and plateau formation corresponding to stable network physical crosslinking. (Results are presented as mean, n = 3).; and (**h**) High-resolution SEM image revealing nanoscale fibrillar and porous architecture of the lyophilized gel. Scale bar: (**h**) 1 µm.

Native omental tissue exhibited its three-dimensional vascularized structure embedded in adipose-rich stroma. (Figure 2a). Following decellularization, the wet ECM retained the overall fibrous morphology of the native tissue but became markedly translucent and more fragile in handling, indicating effective removal of cellular components and lipids while preserving the extracellular scaffold (Figure 2b). Freeze-drying of the decellularized ECM resulted in a thin, scaffolding material with reduced volume and mass (Figure 2c), which was then mechanically milled into a uniform, fine white powder (Figure 2d). The powdered ECM provided a readily storable intermediate form suitable for reproducible batch-to-batch hydrogel fabrication. The ECM powder was enzymatically digested in acidic conditions to produce a clear, homogeneous pre-gel solution (Figure 2e). This pre-gel remained in a liquid state at low temperature, enabling easy handling and injection. Upon neutralization and incubation at 37 °C, rapid physical crosslinking occurred, resulting in a stable hydrogel (Figure 2f). Rheological analysis further confirmed the hydrogel’s thermoresponsive gelation: upon exposure to physiological temperature, the complex modulus exhibited a sharp rise within the first 600 s, followed by a plateau phase, indicating the establishment of a stable cross-linked network (Figure 2g). The final complex modulus values were within the range previously reported for soft tissue-mimicking hydrogels, suggesting suitability for cardiac tissue engineering applications [19]. This sol–gel transition was indicative of preserved fibrillar self-association capacity of the ECM components following processing. High-resolution scanning electron microscopy (SEM) revealed that the ECM-based hydrogel possessed a nanoscale fibrillar network resembling native collagen ultrastructure, with a highly porous and interconnected architecture (Figure 2h). This 3D structure constitutes a supportive microenvironment for cells and nutrient diffusion [44].

### 3.2. Preparation of iPSC-Loaded ECM Hydrogels and Cardiomyocyte Differentiation in 3D

**Figure 3:**
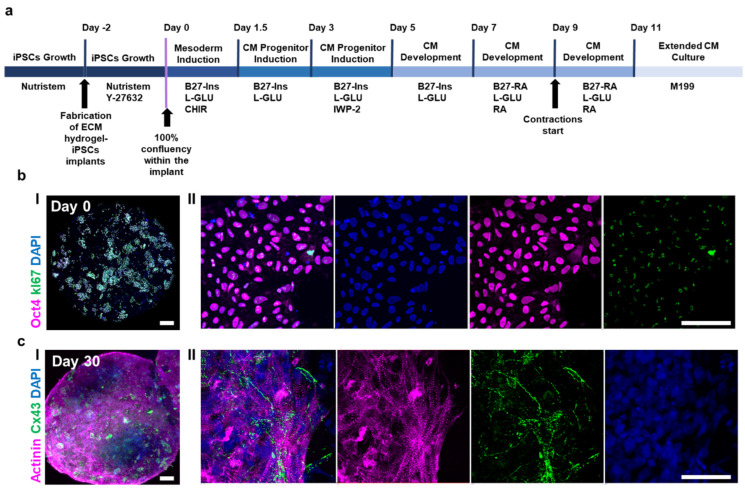
3D cardiac differentiation of iPSC-loaded ECM-based hydrogels. (**a**) Schematic timeline of the differentiation process, starting with the expansion of human iPSCs within ECM hydrogel until confluency, followed by stage-specific induction toward cardiomyocytes. (**b**) Representative immunofluorescence images of iPSCs at day 0 showing strong expression of Oct4 (purple) and high proliferative activity indicated by Ki67 (green), with nuclei counterstained with DAPI (blue). (**c**) By day 30, differentiated cells exhibited elongated morphologies and expressed α-actinin (purple), demonstrating organized contractile structures, and connexin 43 (green), indicating the formation of gap junctions for electrical coupling. Scale bars: (**b**): (**I**) 100 μm, (**II**) 50 μm. (**c**): (**I**) 100 μm and (**II**) 50 μm.

To explore the potential of the ECM-based hydrogel as both a structural scaffold and a bioactive niche, we next generated cardiac tissues by incorporating human induced pluripotent stem cells (iPSCs) directly into the 3D matrix, allowing the entire cardiac differentiation process to occur within the 3D microenvironment (Figure 3a). iPSCs were introduced into the hydrogel two days prior to induction, allowing them to adapt to the 3D microenvironment, establish cell–cell and cell–matrix interactions, and expand to full confluency. At day 0, prior to differentiation, cells retained strong expression of the pluripotency marker Oct4 and exhibited a high proliferation index, as indicated by Ki67 staining, confirming that the hydrogel preserved their stemness and proliferative capabilities (Figure 3b).

A series of stage-specific cues designed to mimic embryonic cardiac development was used to differentiate iPSCs into cardiomyocytes over the course of 11 days. Initial signaling activation drove the cells toward mesodermal lineage commitment, followed by modulation of pathways that favored cardiac progenitor specification. In the later stages, cues promoting sarcomeric assembly, metabolic maturation, and electrophysiological connectivity facilitated the transition into functional cardiomyocytes. Throughout the process, the surrounding ECM hydrogel provided not only structural support but also biochemical and mechanical signals reminiscent of the native cardiac extracellular environment. As previously demonstrated by our group, cells encapsulated in ECM-based hydrogel over the course of differentiation secrete ECM components and soluble factors that can interact with the original ECM fibers [39]. By day 9, spontaneous and coordinated contractions emerged within the implants, indicating successful acquisition of the functional cardiac phenotype (Video S1).

After 30 days of functional maturation, immunostaining revealed abundant expression of α-actinin, indicating the formation of organized contractile fibers, and connexin 43 (Cx43), reflecting the development of gap junctions for electrical coupling between cells (Figure 3c). Cells exhibited elongated and aligned structures, forming an interconnected network across the hydrogel. Nuclei staining confirmed uniform cell distribution throughout the construct. Together, these findings demonstrate that. The ECM hydrogel supported the formation of structurally organized and functionally competent cardiac tissues, characterized by coherent sarcomeric assembly, electrical coupling, and predictable drug responsiveness. Although no direct comparison to 2D differentiation was performed in this study, the structural organization and coherent functional responses observed here are consistent with prior reports demonstrating the advantages of ECM-based 3D environments for cardiac tissue development.

### 3.3. Drug Screening of Cardiac iPSC-Derived Implants at Day 30 of Cultivation

**Figure 4:**
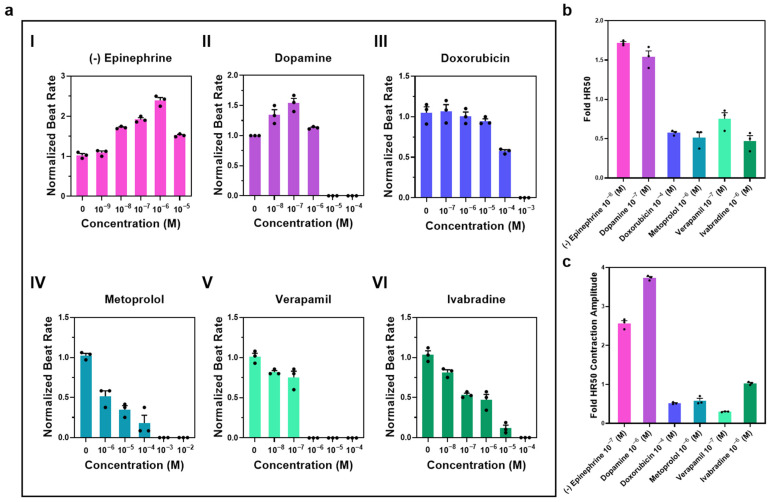
Drug responsiveness of iPSC-derived cardiac tissues at day 30 of cultivation. Beating tissues on day 30 of cultivation were exposed to a panel of cardioactive and cardiotoxic compounds to assess functional responses. (**a**) Concentration–response curves for (**I**) (-)epinephrine, (**II**) dopamine, (**III**) doxorubicin, (**IV**) metoprolol, (**V**) verapamil, and (**VI**) ivabradine, showing the normalized beat rate relative to baseline and revealing the dose-dependent effects of each drug. The half-maximal heart rate change (HR_50_) was calculated for each compound from these curves. (**b**) Comparative analysis of fold-change in HR_50_ for beat rate at the selected concentration of each drug, showing the stimulatory effects of epinephrine and dopamine, the suppressive effects of metoprolol, verapamil, and ivabradine, and the moderate rate reduction induced by doxorubicin. (**c**) Corresponding fold-change in contraction amplitude at HR_50_, illustrating that positive inotropes enhanced amplitude, while negative inotropes and doxorubicin markedly reduced it. Results are presented as mean ± SEM, n = 3. Each sample was individually normalized to its initial value.

Exposure of day 30 cardiac iPSC-derived cardiac tissues to a panel of cardioactive and cardiotoxic agents revealed drug-specific, concentration-dependent modulation of contractile behavior (Figure 4a). Brightfield recordings from an inverted microscope were analyzed to quantify the normalized beat rate for each compound across multiple concentrations, allowing determination of the half-maximal heart rate response (HR_50_). HR_50_ was used as a data-driven normalization point to standardize downstream analyses across compounds with different potency ranges. This metric is not intended to represent a uniform physiological state but rather provides a consistent reference to which secondary functional parameters such as contraction amplitude and calcium handling can be compared. Similar ECₓ normalization strategies are widely used in functional screening studies [28,45]. The β-adrenergic agonist (-)epinephrine elicited the expected dose-dependent acceleration in beat rate, consistent with its role in stimulating β_1_-adrenergic receptors to increase intracellular cAMP and enhance pacemaker current (Figure 4(aI)). Similarly, dopamine produced a biphasic response with rate acceleration at intermediate doses, in line with its β_1_-receptor activation, followed by a decline at higher concentrations, potentially reflecting toxic or desensitizing effects (Figure 4(aII)). In contrast, the anthracycline chemotherapeutic doxorubicin induced a progressive, concentration-dependent reduction in beat rate, as anticipated from its mitochondrial and oxidative stress-mediated cardiotoxicity (Figure 4(aIII)).

Negative chronotropic agents displayed trends consistent with their pharmacological profiles: the β_1_-selective blocker metoprolol markedly reduced beat rate in a dose-dependent manner by antagonizing adrenergic stimulation (Figure 4(aIV)); the L-type calcium channel blocker verapamil slowed rate by reducing calcium influx required for pacemaker cell depolarization (Figure 4(aV)); and the IF channel inhibitor ivabradine decreased rate by directly suppressing the sinoatrial pacemaker current (Figure 4(aVI)). Importantly, the clear and selective response to Ivabradine functionally suggests the presence of functional pacemaker-like cells within the constructs, as the 3D differentiation process yields a heterogeneous cardiac cell population that includes sinoatrial node-like cells known to be sensitive to this compound. These findings are consistent with common functional inference approaches used in 3D iPSC-derived tissues [42,43,46]. Such heterogeneity is beneficial for drug-screening applications, as it more closely reflects the complex cellular environment of native myocardium.

HR_50_ values derived from these dose–response curves were then used to standardize drug concentrations for direct comparison. At HR_50_, (-)epinephrine and dopamine significantly increased fold HR_50_ for beat rate relative to baseline, whereas metoprolol, verapamil, and ivabradine each resulted in marked reductions, and doxorubicin caused a moderate yet consistent decrease (Figure 4b).

Assessment of contraction amplitude at HR_50_ revealed that positive chronotropic agents not only increased pacing frequency but also augmented contractile force, consistent with β-adrenergic enhancement of calcium cycling. Conversely, metoprolol, verapamil, and ivabradine significantly suppressed contraction amplitude, corresponding to their inhibition of calcium handling or pacemaker currents. Doxorubicin also reduced contractile amplitude, reflecting its cumulative cardio-depressant effects (Figure 4c).

When compared to reported human plasma or tissue concentrations, the HR_50_ values derived from our engineered iPSC-cardiac tissues generally fell within, or modestly above, physiological or therapeutic ranges observed in vivo. For example, the HR_50_ for epinephrine and dopamine was in the sub-micromolar range, comparable to their circulating levels during sympathetic activation (0.3–1 µM for epinephrine and 0.1–1 µM for dopamine), indicating that the model recapitulates physiological β_1_-adrenergic sensitivity [23,33,47,48]. Similarly, the inhibitory HR_50_ concentrations for metoprolol and verapamil (in the tens to low hundreds of nM) approached their reported therapeutic plasma levels (0.3–0.9 µM and 0.5–2 µM, respectively), reflecting pharmacological sensitivity consistent with near-physiological responsiveness [36,49,50,51]. The HR_50_ for ivabradine corresponded well with clinically relevant exposures (0.2–0.5 µM) [52,53], while doxorubicin produced half-maximal suppression near its therapeutic plasma concentration (0.05–0.1 µM), consistent with its known cardiotoxic threshold [21,54]. These parallels suggest that the 3D tissue model yields pharmacological sensitivity more comparable to human tissue exposure than conventional 2D monolayers, which typically require higher HR_50_ values due to limited maturation and intercellular coupling. Furthermore, at HR_50_, the correlation between contraction amplitude and chronotropic modulation mirrored in vivo cardiac performance, positive chronotropes enhanced both frequency and force, whereas negative chronotropes and toxic agents reduced them proportionally. Overall, the observed functional responses of the engineered tissues not only align with each drug’s known mechanism of action but also exhibit sensitivity ranges approaching in vivo relevance.

### 3.4. Integrated Electrophysiology and Calcium Imaging of Drug-Induced Cardiomyocyte Modulation

**Figure 5:**
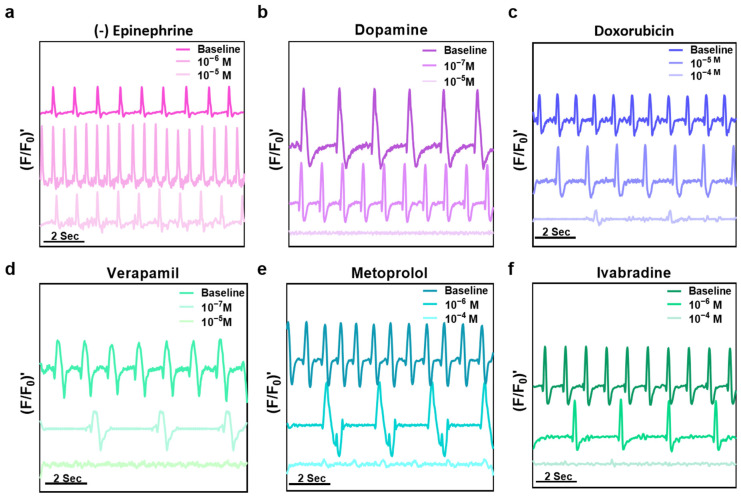
Calcium imaging analysis of drug-induced modulation of cardiomyocyte function. Representative calcium transients, recorded at baseline, the half-maximal heart rate response (HR_50_), and a supraphysiological concentration (>HR_50_). (**a**) (-)-Epinephrine—baseline (0 µM), HR_50_ (1 µM), exceeding (10 µM). Increased transient amplitude and frequency at HR_50_, followed by desensitization at exceeding concentration. (**b**) Dopamine—baseline (0 µM), HR_50_ (0.1 µM), exceeding (10 µM). Produced moderate increases in amplitude and frequency, consistent with mixed receptor activation. (**c**) Doxorubicin—baseline (0 µM), HR_50_ (10 µM), exceeding (100 µM). Strong suppression of amplitude and frequency already at HR_50_, with further impairment at exceeding concentration, reflecting cardiotoxic effects. (**d**) Verapamil—baseline (0 µM), HR_50_ (0.1 µM), exceeding (10 µM). Reduced transient amplitude and slowed frequency in a dose-dependent manner. (**e**) Metoprolol—baseline (0 µM), HR_50_ (1 µM), exceeding (100 µM). Selectively reduced transient frequency with little change in amplitude. (**f**) Ivabradine—baseline (0 µM), HR_50_ (1 µM), exceeding (100 µM). Strongly reduced transient frequency while preserving amplitude.

Following the contractility-based drug screening, in which the beat rate and contraction amplitude responses to each compound were quantified at varying concentrations to determine the half-maximal heart rate response (HR_50_), the analysis was expanded to investigate underlying calcium handling dynamics. Due to the versatility of mechanisms among the compounds in the panel, calcium imaging analysis was utilized to detect potential alterations in calcium channel activity. Calcium imaging-based measurements (Figure 5) revealed distinct patterns of cardiomyocyte functional modulation for each pharmacological treatment under baseline conditions, at HR_50_, and at a supraphysiological concentration exceeding HR_50_. Some variability was observed in baseline calcium transient morphology, which is expected in 3D iPSC-derived constructs due to inherent differentiation heterogeneity; importantly, all constructs exhibited consistent, directionally appropriate responses to pharmacological stimuli. For the β-adrenergic agonist (-)-epinephrine, calcium transient amplitude and frequency both increased at HR_50_, consistent with the expected enhancement of L-type calcium channel opening and sarcoplasmic reticulum calcium release via β_1_-receptor stimulation. At the concentration of (-)-epinephrine exceeding HR_50_, calcium transient amplitude and frequency decreased indicating receptor desensitization or calcium handling saturation (Figure 5a). Dopamine exhibited a similar but less pronounced pattern, in line with its mixed β_1_-adrenergic and dopaminergic receptor activation profile, producing moderate positive inotropic and chronotropic effects at HR_50_ (Figure 5b). Doxorubicin, a cardiotoxic anthracycline, caused a reduction in transient amplitude and frequency already at HR_50_, matching the expected impairment in calcium cycling due to oxidative stress and interference with sarcoplasmic reticulum function, which became more severe at higher doses (Figure 5c). Verapamil, a calcium channel blocker, markedly diminished transient amplitude and slowed frequency in a dose-dependent manner, reflecting its L-type calcium channel inhibition (Figure 5d). The β-blocker metoprolol reduced transient frequency at HR_50_ with minimal change in amplitude, consistent with its antagonism of β_1_-receptors and negative chronotropic effect (Figure 5e). Ivabradine, a selective funny current (If) inhibitor, produced a pronounced decrease in transient frequency while largely preserving amplitude, in agreement with its mechanism of pacemaker current inhibition without directly altering calcium release (Figure 5f).

**Figure 6:**
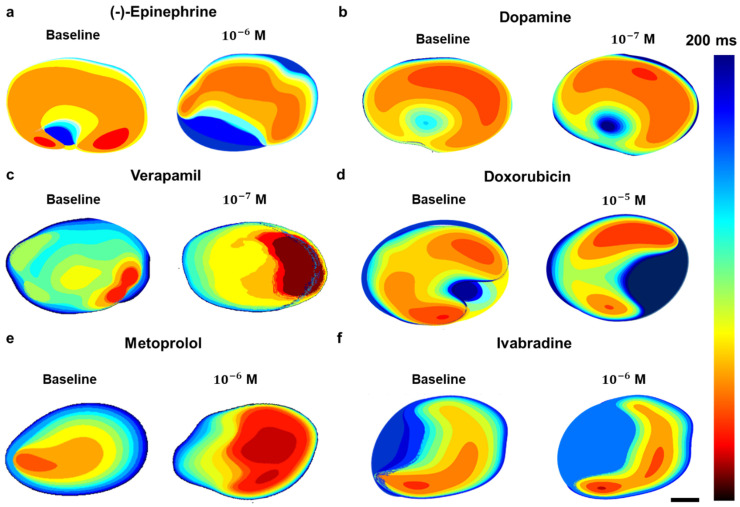
Conduction velocity mapping of cardiac tissue constructs before and after drug exposure at HR_50_ concentrations. Isochronal maps illustrate the spatiotemporal propagation of electrical activity across the engineered cardiac tissues under baseline conditions (left panels) and following exposure to each compound at its half-maximal heart rate response (HR_50_) concentration (right panels). (**a**) (-)-Epinephrine (10^−6^ M), (**b**) Dopamine (10^−7^ M), (**c**) Verapamil (10^−7^ M), (**d**) Doxorubicin (10^−5^ M), (**e**) Metoprolol (10^−6^ M), and (**f**) Ivabradine (10^−6^ M). Color scale indicates activation time in milliseconds, with warmer colors representing earlier activation and cooler colors later activation. Scale bar: 200 µm.

Analysis of conduction velocity maps (Figure 6) revealed distinct, drug-specific modulation of electrical propagation across the engineered cardiac constructs, with indicative regions in each heat map highlighting characteristic conduction patterns. For (-)-epinephrine (10^−6^ M, HR_50_), the heat map (Figure 6a) showed tightly clustered isochrones and a sharply advancing activation wavefront originating from the lower-left quadrant, reflecting a global acceleration of conduction. This rapid propagation is consistent with β_1_-adrenergic stimulation, which enhances sodium current availability and strengthens gap junction coupling, leading to a more synchronized activation pattern. Dopamine (10^−7^ M) exhibited a similar but milder profile (Figure 6b), with moderate narrowing of isochronal lines particularly visible along the central longitudinal axis, indicative of partially enhanced excitability through combined β_1_ and dopaminergic receptor activity.

In contrast, verapamil (10^−7^ M) produced a pronounced slowing of conduction (Figure 6c), with widely spaced isochrones and delayed activation zones especially apparent in the right region of the tissue. This pattern corresponds to L-type calcium channel blockade, resulting in prolonged action potential upstroke and impaired cell-to-cell current transfer. Doxorubicin (10^−5^ M) caused marked spatial heterogeneity in conduction (Figure 6d), with patchy low-velocity zones, most evident in the upper and lower peripheral sectors. These irregular patterns reflect disrupted ion homeostasis and compromised intercellular coupling, consistent with the drug’s arrhythmogenic cardiotoxicity. With metoprolol (10^−6^ M), conduction velocity was modestly reduced (Figure 6e), as reflected by slightly expanded isochronal intervals across the field while maintaining overall uniform propagation. This likely stems from β_1_-blockade-mediated decreases in excitability without direct sodium channel inhibition. Finally, ivabradine (10^−6^ M) primarily reduced beat frequency while preserving homogeneous propagation (Figure 6f), evident in the uniform isochronal distribution and steady activation front. This pattern aligns with If current inhibition, which slows pacemaker depolarization without perturbing myocardial conduction velocity. These activation maps reveal drug-specific alterations in propagation patterns, providing meaningful functional insight even without absolute numerical conduction velocity measurements. Complementary dynamic heat map videos (Videos S2–S13) corroborate these observations, visualizing spatial–temporal modulation of calcium transients, conduction velocity, contraction amplitude, and beat rate at baseline and HR_50_ conditions. Collectively, these results demonstrate the platform’s capacity to capture drug-specific electrophysiological signatures with spatial precision, underscoring its translational potential as a predictive preclinical model for cardiotoxicity and drug screening.

## 4. Discussion

The present study demonstrates the feasibility of engineering extracellular matrix (ECM)-based cardiac tissues, integrated with human iPSCs, and functionally matured into spontaneously beating cardiomyocyte constructs for cardiotoxicity screening. By combining natural biomaterials, pluripotent stem cell technology, and integrative functional assays, we establish a versatile platform that advances both regenerative medicine applications and preclinical pharmacology.

The successful decellularization and processing of omental tissue into a thermoresponsive hydrogel (Figure 2 and Figure 3) highlight the advantages of ECM-based matrices as supportive niches for early cardiac lineage specification. The preserved biochemical and mechanical cues within native ECM are known to influence cell fate and maturation, and our findings support this paradigm: the 3D constructs exhibited coherent sarcomeric organization, synchronous contraction, and predictable pharmacological responses. Unlike synthetic hydrogels, which often lack biological complexity, the ECM-derived scaffold provides a physiologically relevant microenvironment that more closely mimics native cardiac tissue.

A central innovation of this work is the direct 3D differentiation of iPSCs within the ECM hydrogel, differing from the more common strategy of performing 2D monolayer differentiation followed by reseeding into a 3D scaffold. While 2D protocols efficiently generate cardiomyocytes, they often yield immature phenotypes and limited structural integration after transfer to 3D environments. By initiating differentiation directly in a 3D ECM context, our approach more closely resembles embryonic cardiogenesis, where cells differentiate, interact, and self-organize within a three-dimensional matrix. Although no direct comparison to 2D monolayer differentiation was performed here, the structural organization and functional responsiveness observed in our constructs align with prior studies showing the advantages of ECM-based 3D environments for cardiac tissue development.

The constructs also naturally developed heterogeneous cardiac populations, which is expected for 3D ECM-rich differentiation environments and supported by our prior work [13,38,40,41]. Functional heterogeneity was reflected in the spontaneous beating behaviors and calcium transient variability observed across constructs. Importantly, the selective chronotropic response to ivabradine provides functional evidence for the presence of pacemaker-like cells, consistent with commonly accepted inference approaches in 3D iPSC-derived cardiac tissues. Such heterogeneity is not only expected but beneficial for drug-screening applications, as it more accurately reflects the multicellular complexity of native myocardium.

Drug exposure experiments (Figure 4 and Figure 5) further validated the functionality and sensitivity of these constructs. The observed drug-induced modulations in beat rate, contraction amplitude, and calcium transients were consistent with established pharmacological mechanisms. For example, the positive inotropic and chronotropic effects of epinephrine and dopamine aligned with their β-adrenergic stimulation profiles, whereas verapamil and metoprolol produced the expected reductions in calcium transients and beat frequency through L-type calcium channel blockade and β-blockade, respectively. Importantly, the cardiotoxic effects of doxorubicin were faithfully recapitulated in our model, with impaired calcium handling emerging at clinically relevant concentrations, consistent with previous reports of anthracycline-induced cardiotoxicity [55,56]. These results confirm the validity of the model for systemic drug testing and could support future applications for cardiotoxicity screening. Similarly, ivabradine selectively reduced beat frequency without altering amplitude, in agreement with its mechanism as an If channel inhibitor [37,57]. These results not only confirm the predictive validity of the platform but also underscore its sensitivity to detect both therapeutic and adverse drug effects.

Conduction mapping derived from calcium imaging revealed drug-specific alterations in activation patterns. While calcium imaging provides an indirect approximation of conduction velocity, it remains widely used for 3D engineered tissues when voltage mapping is limited by optical properties or tissue thickness. Accordingly, we interpreted conduction changes qualitatively and semi-quantitatively, recognizing that precise velocity measurements would require voltage-sensitive dyes or microelectrode technologies.

HR_50_ was used as a data-driven normalization point to align downstream functional comparisons across drugs with distinct potency profiles. This metric is not intended to imply equivalent physiological states among compounds, but rather to anchor analyses at a consistent chronotropic modulation point, analogous to EC_x_/IC_x_ normalization strategies commonly used in pharmacological studies.

When viewed in the context of prior literature, our findings suggest that ECM-based iPSC-cardiac constructs can bridge the gap between conventional 2D cardiomyocyte cultures and animal models. Unlike 2D systems, which often lack physiological relevance, our constructs preserve tissue-level interactions and the dynamics of excitation–contraction coupling. Concurrently, compared to animal models, they offer a human-specific platform that bypasses interspecies variability in drug response. This positions the system as a promising tool for both personalized medicines, by using patient-derived iPSCs, and broader generalizable drug discovery pipelines.

Despite these promising results, several limitations should be acknowledged. The constructs should be further investigated for expressing adult cardiomyocyte electrophysiological maturity and for detailed mapping of cardiac subtypes. Other future aspects should explore the effect of chronic drug exposure, functionality post compounds washout, and arrhythmogenic marker expressions such as APD90, EADs, and DADs. Additionally, although ECM hydrogels produced using our established protocol have demonstrated reproducibility, biological matrices inherently introduce batch-to-batch variability. Recognizing these limitations helps contextualize the scope of the current work and identifies important areas for future development.

Future directions for this research include optimizing long-term culture stability for the maturation of constructs, integrating vascularization strategies to support the formation of thicker tissue and physiologically relevant drug diffusion assays, and expanding the drug screening repertoire to include arrhythmogenic and metabolic modulators. The integration of advanced imaging modalities and computational modeling could further enhance the predictive power of the platform. Moreover, the ability to scale this approach for high-throughput applications may accelerate its translation toward preclinical testing and safer pharmacology. Importantly, recent advances in 3D bioprinting now allow the fabrication of vascularized cardiac tissues with perfusable networks, providing an opportunity to study how drugs are delivered through the vasculature and affect parenchymal cells in a more physiologically accurate context [40,41,58,59,60]. This capability could enable more precise assessment of pharmacokinetics and localized tissue responses, bridging a critical gap between conventional in vitro assays and in vivo outcomes.

## 5. Conclusions

In summary, this study establishes a novel strategy for direct differentiation of iPSCs within a decellularized ECM hydrogel that yields to structurally and functionally mature cardiac tissues that faithfully reproduce expected drug responses and cardiotoxic effects. This strategy addresses limitations of 2D differentiation and animal models, offering a human-specific, physiologically relevant platform for regenerative medicine, disease modeling, and precision pharmacology.

## Figures and Tables

**Table 1:** Overview of the investigated compounds and their predicted effects on cardiac tissue physiology.

Compound	Primary Mechanism of Action	Beat Rate (Chronotropy)	Contraction Amplitude (Inotropy)	Calcium Transient Propagation
(-) Epinephrine	β_1_-adrenergic receptor agonist; increases cAMP and Ca^2+^ influx [29,33]	Increased	Increased	Faster
Dopamine	β_1_-adrenergic receptor agonist [29,33]	Increased	Increased	Faster
Doxorubicin	Induces oxidative stress and mitochondrial dysfunction; disrupts Ca^2+^ handling [21,30]	Decreased and may become irregular	Decreased	Slower and irregular; may induce arrhythmogenic propagation
Verapamil	L-type Ca^2+^ channel blocker; reduces Ca^2+^ influx [34]	Slightly decreased	Decreased	Slower
Metoprolol	β_1_-adrenergic receptor antagonist; suppresses sympathetic signaling [35,36]	Decreased	Decreased	Slower
Ivabradine	Selective If-channel inhibitor in sinoatrial node [31,37]	Decreased	Largely unchanged	Largely unchanged

## Data Availability

The original contributions presented in this study are included in the article/Supplementary Materials. Further inquiries can be directed to the corresponding author.

## Supplementary Materials

The following supporting information can be downloaded at: https://www.mdpi.com/article/10.3390/pharmaceutics18010018/s1.
